# Swelling and Mechanical Properties of Polyacrylamide-Derivative Dual-Crosslink Hydrogels Having Metal–Ligand Coordination Bonds as Transient Crosslinks

**DOI:** 10.3390/gels7020072

**Published:** 2021-06-15

**Authors:** Louis Debertrand, Jingwen Zhao, Costantino Creton, Tetsuharu Narita

**Affiliations:** 1 Laboratoire Sciences et Ingénierie de la Matière Molle, ESPCI Paris, PSL University, Sorbonne Université, CNRS, F-75005 Paris, France; louisdeb3@gmail.com (L.D.); jingwenzhao1990@gmail.com (J.Z.); 2 Global Station for Soft Matter, Global Institution for Collaborative Research and Education, Hokkaido University, Sapporo 001-0021, Japan

**Keywords:** tough hydrogel, swelling, metal–ligand coordination

## Abstract

Hydrogels that have both permanent chemical crosslinks and transient physical crosslinks are good model systems to represent tough gels. Such “dual-crosslink” hydrogels can be prepared either by simultaneous polymerization and dual crosslinking (one-pot synthesis) or by diffusion/complexation of the physical crosslinks to the chemical network (diffusion method). To study the effects of the preparation methods and of the crosslinking ratio on the mechanical properties, the equilibrium swelling of the dual-crosslink gels need to be examined. Since most of these gels are polyelectrolytes, their swelling properties are complex, so no systematic study has been reported. In this work, we synthesized model dual-crosslink gels with metal–ligand coordination bonds as physical crosslinks by both methods, and we proposed a simple way of adding salt to control the swelling ratio prepared by ion diffusion. Tensile and linear rheological tests of the gels at the same swelling ratio showed that during the one-pot synthesis, free radical polymerization was affected by the transition metal ions used as physical crosslinkers, while the presence of electrostatic interactions did not affect the role of the metal complexes on the mechanical properties.

## 1. Introduction

The mechanical reinforcement of hydrogels is a hot topic in gel science because fracture-resistant hydrogels can find various applications, especially in biomedicine [1,2]. A variety of reinforcement strategies, based on different network architectures and interactions used as crosslinking points, to obtain tough hydrogels have been reported in the literature [3,4,5]. Introducing a dissipative mechanism into the polymer network is one successful method as energy dissipation relaxes stresses and mitigates stress concentration at the crack tip, delaying the propagation of a macroscopic crack [6]. Reversible physical bonds can be used as crosslinkers to break and dissipate energy and reform over shorter or longer times to possibly self-recover the original network. Various types of interactions can be used as reversible physical crosslinks, such as dynamic covalent bonds, hydrophobic interactions, and electrostatic interactions. A wide range of bond lifetimes can also be selected [7,8,9,10]. As an example, nanocomposite hydrogels, composed of polymers such as poly(N-isopropylacrylamide) and inorganic particles such as laponite and silica, exhibit pronounced energy dissipation due to the physical absorption of the polymer chains onto the nanoparticle surface [6,11]. Coordination bonds with metal ions are a practical option for creating tunable transient crosslinks, and various hydrogels crosslinked by metal–ligand coordination bonds using different metal ions (Fe, Ni, Zn, Rb) and ligands (terpyridine, imidazole) have been reported [12,13,14,15,16].

The lifetime of a metal–ligand coordination bond can be much shorter than the typical observation time, and in recent experiments, such a short-lived crosslink has been found to be efficient for improving toughness [17,18]. Since a short-lived transient crosslink can be rheologically invisible, its practical usefulness was demonstrated in a “dual-crosslink” network, which combined a small amount of a permanent chemical crosslink to a physically crosslinked gel. Short-lived transient crosslinks improved the extensibility at break compared to the corresponding chemical gel [7,17,18]. The presence of permanent crosslinks can prevent plastic terminal flow to allow the systematic characterization of the rheological impact of short-lived physical crosslinks over a wide range of frequencies at varied crosslinking ratios.

It is noteworthy that most of the reported tough dissipative hydrogels with reasonably high water content (>80%) are polyelectrolytes even though the electrostatic attractive interactions are not necessarily used as physical crosslinks. In order to have significant energy dissipation, a large fraction of physical crosslinks is necessary, resulting in not only an increase in the level of gel viscoelasticity, but also a possible decrease in the equilibrium swelling ratio. Fully neutral hydrogels with a high crosslinking ratio may become poorly soluble in water, causing heterogeneities and loss of transparency. Charged groups on networks and their counter-ions, on the other hand, can enhance hydrophilicity and homogeneity. The electrostatic interactions and swelling properties of these tough dissipative hydrogels have not been systematically discussed. How can we systematically compare dissipative hydrogels having different crosslinking degrees and thus different equilibrium swelling ratios? A simple way is to compare the hydrogels at the polymer concentration of preparation. As-prepared hydrogels are good model systems for studying gel properties in the laboratory in air, but their out-of-equilibrium nature may not be very relevant in applications in water, as swelling can occur on contact with a solvent (water). Another gel preparation method to prepare gel samples at equilibrium swelling would be practical.

Two synthesis routes have been commonly used to develop a polymer network having simultaneous chemical and physical crosslinks. Polymerization and dual crosslinking can be performed simultaneously by mixing the monomers and two types of crosslinkers. The obtained as-prepared gels are ready for measurement. This “one-pot” synthesis is simple and fast, and allows good control of the concentration of both the polymer and the physical crosslinks being incorporated. Another method consists of first synthesizing a chemical network of the polymer in water and then incorporating physical crosslinkers into the network through diffusion and complexation. With this “diffusion” method, the obtained gel is at equilibrium swelling. This method is particularly useful when preparation needs to start from a linear polymer (not monomer) since it is generally difficult to introduce a large amount of physical crosslinks before the chemical crosslinks: physical crosslinks are usually formed more rapidly than chemical crosslinks and the obtained physical gel is highly viscous, so further homogenous mixing of the chemical crosslinkers is not practical.

To the best of our knowledge, no systematic study has been conducted on the effects of the preparation methods of the mechanical properties of the resulting dual-crosslink gels. A meaningful comparison of hydrogel mechanical properties can only be carried out at the same water content, and this introduces some constraints: one-pot synthesis allows swelling-ratio control during preparation, while in the diffusion method, dual-crosslink gels are prepared at equilibrium swelling, and any change in the density of the chemical or physical crosslinks causes a change in the equilibrium swelling ratio. Thus to compare dual-crosslink gels prepared by different methods and different crosslinking degrees, it is necessary to develop a systematic methodology to control the equilibrium swelling ratio.

In this paper, we investigated the effects of the preparation methods (one-pot synthesis or diffusion) on the swelling and mechanical properties of model dual-crosslink hydrogels. As previously reported, we used a simple hydrosoluble polymer, copolymerized with *N*-vinylimidazole [18] as a model. Due to imidazole–metal-ion coordination bonds this gel, synthesized by free radical copolymerization, possessed a fast and tunable dynamic, and the ingredients were commercially available and readily soluble in water.

The mechanical properties of the tough hydrogels, obtained by both synthesis methods, were characterized by both small-strain linear rheology and large-strain uniaxial tensile tests. We showed that although the kinetics of the free radical polymerization and the role played by the electrostatic interactions can be influenced by the transition metal ions used as physical crosslinkers, the differences in mechanical properties due to the preparation methods were not very large.

## 2. Results and Discussion

**Figure 1:**
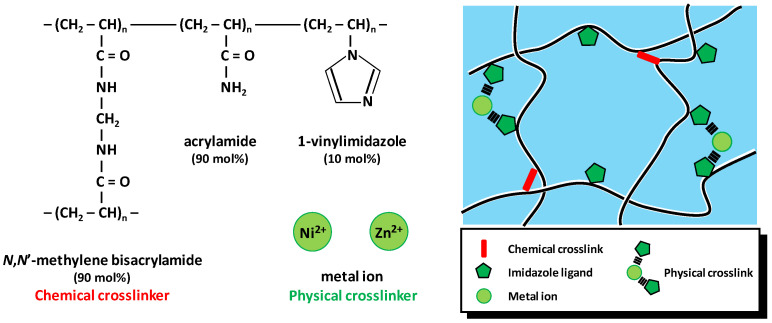
Structure of P(AAm-*co*-VIm)–M^2+^ dual-crosslink gels (M: Ni or Zn).

Our model dual-crosslink gels consisted of neutral poly(acrylamide-*co*-1-vinylimidazole), P(AAm-*co*-VIm), having 10 mol% of VIm. Polyacrylamide was chosen as a commonly used neutral polymer. Imidazole ligands for transient physical crosslinking by metal ligand coordination bonds were introduced by copolymerization with the co-monomer 1-vinylimidazole, which is highly water soluble and commercially available. *N*,*N*’-methylene bisacrylamide was used as chemical crosslinker (0.15 mol% relative to monomer concentration). Various transition metal ions formed coordination complexes with imidazole ligands, and here we show the results with two, Ni^2+^ or Zn^2+^, for tuning the bond’s lifetime. The chemical structure of the gels is schematically shown in Figure 1.

### 2.1. Dual-Crosslink Gels by One-Pot Synthesis

The one-pot synthesis method is a simple way to prepare dual-crosslink gels of variable crosslinking ratios at the same overall polymer concentration. The synthesized gels can also be compared in the as-prepared state. This synthesis method consists of the simultaneous copolymerization and chemical and physical crosslinking; thus, the whole procedure can be carried out in just one reactor. In our study, a chemical gel of poly(acrylamide-*co*-1-vinylimidazole), P(AAm-*co*-VIm), with 10 mol% of VIm (and 0.15 mol% of the chemical crosslinker *N*,*N*’-methylene bisacrylamide) was obtained without a noticeable difference from conventional PAAm chemical gels.

**Figure 2:**
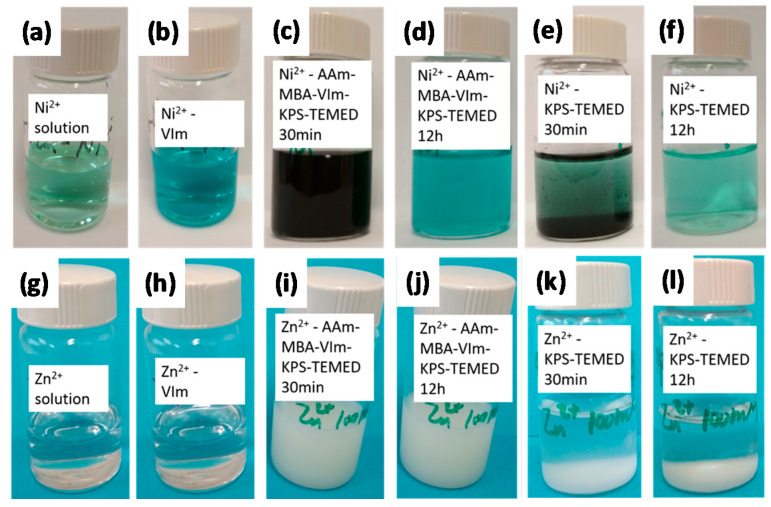
(**a**–**l**) The color and turbidity change during polymerization of P(AAm-*co*-VIm)–M^2+^ dual-crosslink gels (M: Ni or Zn).

The polymerization of the P(AAm-*co*-VIm)-M^2+^ dual-crosslink gel was carried out in the same way as that of the chemical gel, except that the dissolved metal ions Ni^2+^ or Zn^2+^ were used as physical crosslinkers via metal–ligand coordination bonds. During the free radical polymerization process in the presence of Ni^2+^ or Zn^2+^, we observed different behaviors. In Figure 2, we show pictures of the dual-crosslink gel samples having Ni^2+^ (Figure 2d) and Zn^2+^ (Figure 2j) dissolved ions. The P(AAm-*co*-VIm)-Ni^2+^ gel exhibited a blue color and a high transparency 12 h after radical polymerization, while the P(AAm-*co*-VIm)-Zn^2+^ gel showed no color but did show turbidity. In order to understand these results, pictures of control samples are also shown. In Figure 2a, the NiCl_2_ solution (100 mM with no other chemicals for gel synthesis) has a distinctly green color, but the addition of the VIm monomer induced an immediate change to blue (Figure 2b) due to the complexation between Ni^2+^ and the imidazole ligands. This result suggests that the complexes that can serve as physical crosslinkers exist during polymerization and may influence the polymerization and chemical-crosslinking kinetics; therefore, the structure of the final chemical network. When the KPS-TEMED initiator system was introduced in the solution, it became turbid and took on a dark color (Figure 2c); however, the color changed to almost a transparent blue during polymerization at room temperature (Figure 2d). Since the same mixture without Ni^2+^ (the corresponding P(AAm-*co*-VIm) chemical gel) did not show any change in turbidity or color (picture not shown), we concluded that the interactions between the persulfate radical and Ni^2+^ were involved. For the preparation of P(AAm-*co*-VIm)-Zn^2+^ gel, the ZnCl_2_ solution (100 mM) has no color (Figure 2g) even after complexation between Zn^2+^ and the imidazole ligands (Figure 2h). After adding KPS-TEMED, however, the solution became turbid and white (Figure 2i), and no visible color or turbidity change occurred during polymerization (Figure 2j).

To explain the change in turbidity, a series of complementary experiments was carried out, and the reaction between KPS and metal ions was found to be responsible. Figure 2a,e,f show the reaction between KPS and the Ni^2+^ solution. Once KPS and TEMED were added (generating persulfate radicals), a black precipitate appeared in the Ni^2+^ solution, (Figure 2e). Though it was not analyzed, its appearance suggested nickel(III) oxide or nickel oxide hydroxide NiOOH given the interaction between Ni^2+^ and the strongly oxidizing radical produced by KPS [19]. The precipitate mostly disappeared after several hours (Figure 2f) due to the slightly acidic environment (pH = 6.8). This could explain the appearance and disappearance of turbidity during polymerization. For the case of Zn^2+^, a white precipitate was observed after adding KPS and TEMED (Figure 2k), presumably Zinc oxide (ZnO). Unlike the black precipitate in the Ni^2+^ solution, this one did not disappear (Figure 2l), which is the reason that the P(AAm-*co*-VIm)-Zn^2+^ gel remained white.

The amount of precipitate changed with the KPS concentration (pictures not shown). It should be noted that the pH of the solution was sensitive to the KPS concentration and that the strength of the metal–ligand coordination bond studied here was pH dependent. At high pH, the solubility of these transition metal ions decreased, and at low pH the protonation of the imidazole ligands competed with the coordination. Therefore, the available pH range where complexation dominated was approximately between 5 and 7. Under the conditions described in Figure 2, the concentration of KPS (6 mM) was much lower than that of the metal ions (100 mM), and despite the undesired oxidation reaction with the ions, part of the radicals was consumed to initiate polymerization. Thus the majority of the metal ions were not oxidized and served as physical crosslinks as expected. The theoretical maximal concentration of ZnO in the presence of 6 mM of KPS is about 1 g/L; thus, the effect of the white ZnO precipitate on the mechanical properties of the P(AAm-*co*-VIm)-Zn^2+^ gel is negligible though there might have been interactions between the particles and the ligands [20]. However, the influence of the oxidation can be important when the metal ion concentration is lower. As a further example, we tested CuCl_2_ as a metal-ion provider, and no gelation occurred due to the strong interactions between the persulfate radicals and Cu^2+^. These results suggest that free radical polymerization needs to be finely tuned in the one-pot synthesis in this dual-crosslink hydrogel system. It had been previously shown that during the one-pot synthesis of a nanocomposite gel, interactions between the radicals and silica nanoparticles affected polymerization [21].

### 2.2. Dual-Crosslink Gels by the Diffusion Method

The one-pot synthesis allowed us to prepare the P(AAm-*co*-VIm)-M^2+^ dual-crosslink gels at a well-defined polymer concentration/swelling ratio. However, for this particular metal–ligand coordination system, the presence of metal ions affected the radical polymerizations, and depending of the metal ion species different behaviors were observed. In this section, we characterize the P(AAm-*co*-VIm)-M^2+^ gels prepared by diffusion. As described in the experimental section, the gels were prepared in three steps. First a free radical polymerization of the chemical gel was conducted in the absence of the metal ions to avoid the precipitates that came from the one-pot synthesis. Then, the chemical gel swelled to equilibrium to remove unreacted monomers, and finally the physical crosslinkers (metal ions) were incorporated by diffusion. Contrary to the one-pot method, during the second and third steps the gels swelled to equilibrium, and the final polymer concentration depended on the crosslinking ratio. We evaluated the swelling behavior of the dual-crosslink gels.

#### 2.2.1. Absorption of Ni^2+^ Ions

**Figure 3:**
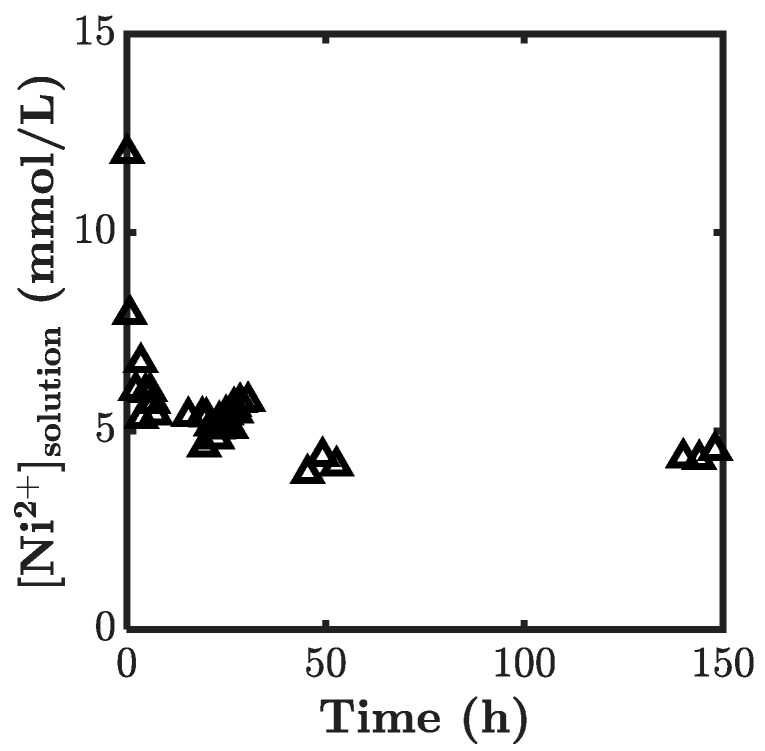
Decrease of Ni^2+^ concentration in the solution due to uptake by a chemical gel as a function of time.

To quantify the process of incorporating the metal ions (used as physical crosslinkers) into the chemical gel by the diffusion method, we first studied the kinetics of ion absorption into the chemical gel to determine the equilibration time. A sheet of P(AAm-*co*-VIm) chemical gel at swelling equilibrium in water was immersed in a solution of NiCl_2_, and the change in the Ni^2+^ concentration because of the gel absorption was monitored using a UV-visible spectrometer (absorption of Ni^2+^ in water at 395 nm). Figure 3 shows the time-dependent Ni^2+^ concentration in the solution. We clearly see in that it decreased with time to reach at an equilibrium state at around 50 h of diffusion. We then chose three days of equilibration to ensure that the system was indeed at equilibrium. The order of magnitude of the diffusion coefficient was estimated from diffusion theory and from the knowledge of the equilibration time and sample thickness. With an equilibration time of 50 h, we found *D* ~ 10^−12^ m^2^/s, much lower than the diffusion coefficient of the ion in water, and in a swollen gel on the order of 10^–9^ m^2^/s [22]. This low *D* value is due to the absorption of part of the ions to the polymer chains, which decreased the concentration of mobile ions diffusing into the gel [23].

#### 2.2.2. Swelling of the Dual-Crosslink Gels and Effect of NaCl

**Figure 4:**
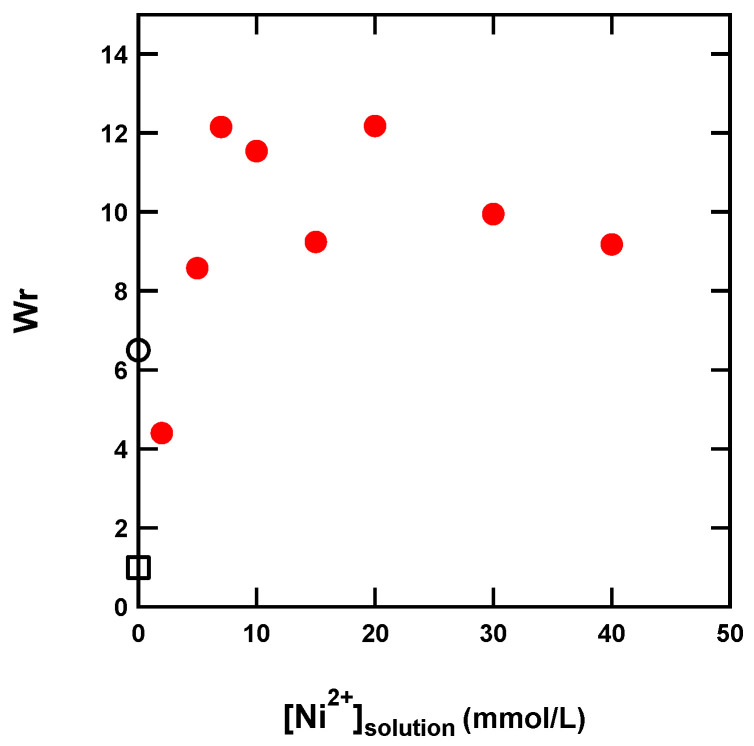
Swelling ratio of dual-crosslink hydrogels in Ni^2+^ solutions of different concentrations (filled circles). The value of the chemical gel as prepared is 1, plotted as an open square, and that of the chemical gel at equilibrium swelling in water (corresponding to a weight percentage in water of around 97.5%) is indicated by the open circle at about 6.5.

Here, we evaluated the swelling properties of the dual-crosslink gel prepared by the diffusion method. As a swelling ratio, we used the weight ratio *W*_r_ between the gel at equilibrium and the as-prepared gel. The results are plotted in Figure 4. The dependence of the equilibrium swelling ratio on the Ni^2+^ concentration is complex. The swelling ratio first decreased to about 4.4 at [Ni^2+^] = 2 mM, then increased to 9–12, suggesting that the Ni^2+^ ions had two opposite effects on the swelling properties of the dual-crosslink gel. The complexes formed between the Ni^2+^ ions and the imidazole ligands on the chemical gel served as additional crosslinks that decreased the *W*_r_. Simultaneously, Ni^2+^ ions introduced positive charges to the neutral chemical gel (at the given pH of 6.5, protonation of imidazole was limited), and the counter-ions (Cl^−^) increased the osmotic pressure of the gel and increased the *W*_r_. At low Ni^2+^ concentrations, the crosslinking effect was dominant and the gel deswelled, but with increased Ni^2+^, the charge effect became dominant and the swelling ratio increased. The same behavior was observed for the dual-crosslink gels made from neutral poly(vinyl alcohol) and borate ions, which brought negative charges (and counter-ions) to the network [24]. 

**Figure 5:**
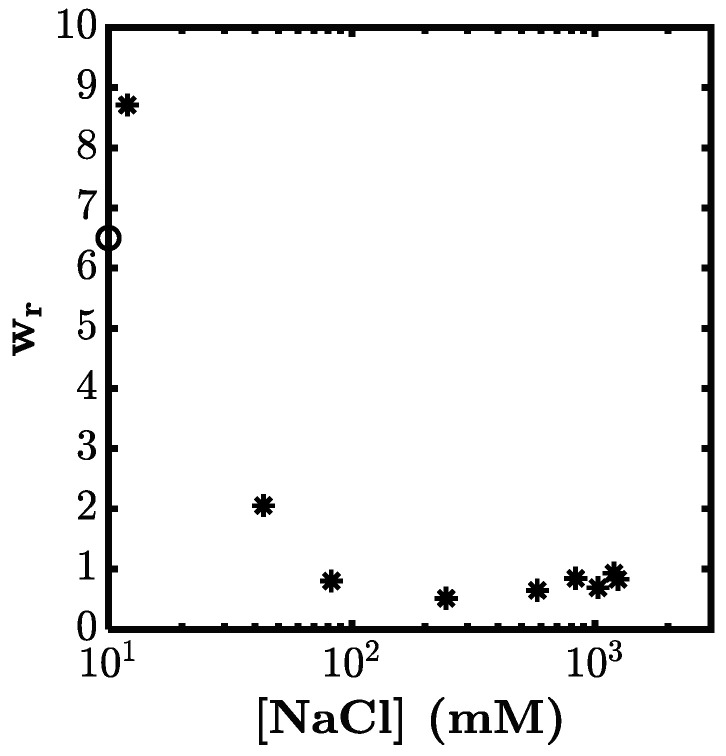
Swelling ratio of the P(AAm-*co*-VIm)-Na^2+^ dual-crosslink gel with [Ni^2+^] = 5 mM as a function of salt concentration [NaCl]. The open circle represents the swelling ratio of the chemical gel in distilled water (without NiCl_2_ or NaCl).

In the context of gel toughening, the observed high swelling was not desirable as the obtained gels became more fragile and brittle. To prevent charge-induced swelling and to compare the mechanical properties at the same *W*_r_, it was necessary to adjust the swelling ratio to that of the as-prepared gel. Here we showed the effect of added salt (NaCl) to decrease the osmotic pressure and to deswell the gel to the swelling ratio of 1 by the crosslinking effect. In Figure 5, we show how the *W*_r_ depended on NaCl concentration ([Ni^2+^] = 5 mM). The swelling ratio *W*_r_ showed a pronounced NaCl concentration dependence. As seen in the figure, the dual-crosslink gel deswelled in the presence of several tens of mM of NaCl, to level off at about *W*_r_ = 1. This result indicated that the swelling ratio could be controlled by the addition of salt to reach the swelling ratio of the as-prepared gels.

**Figure 6:**
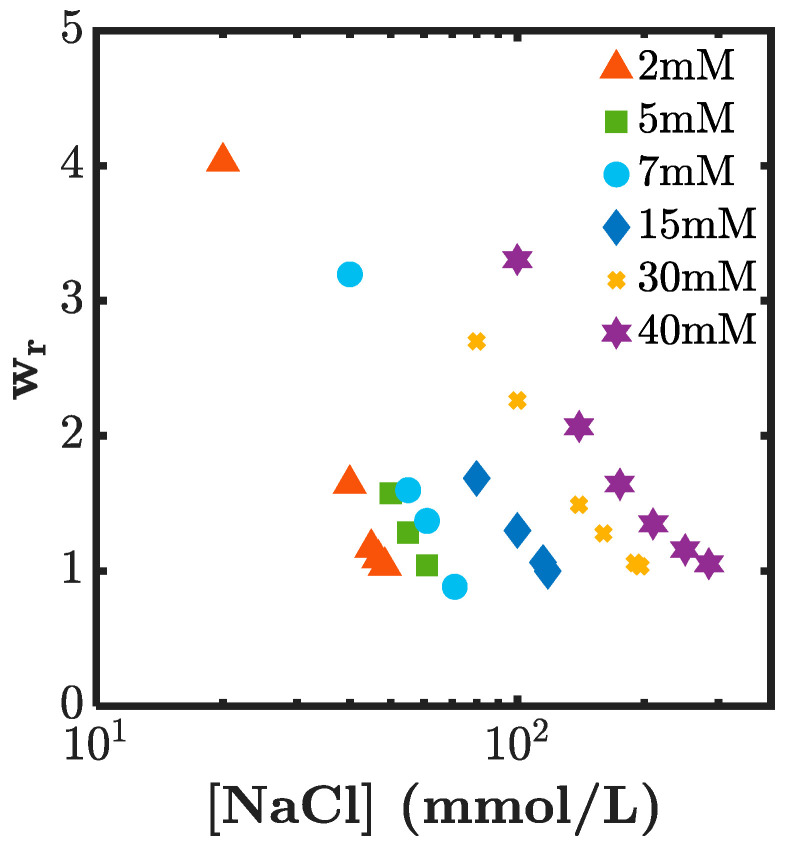
Swelling ratio *W*_r_ of the dual-crosslink hydrogel for different initial concentrations in NiCl_2_, versus the NaCl concentration in the solution.

The same experiments were performed for different concentration values of Ni^2+^ and are shown in Figure 6. For low Ni^2+^ concentrations, we did not expect the swelling ratio to decrease to 1 easily (due to low physical crosslinking and low charge density), but we found that for all tested Ni^2+^ concentrations, the swelling ratio could be adjusted to 1 and NaCl values increased with Ni^2+^ concentration to reach *W*_r_ = 1. The value of NaCl necessary for the swelling ratio adjustment ranged between about 50 and 300 mM. These values should be comparable to the concentration of charges on the dual-crosslink gel, or equivalently to the concentration of the bound Ni^2+^.

The concentration of the imidazole ligands in the feed was 200 mM. Bicomplexes of one Ni^2+^ ion and two imidazole ligands served as crosslinks, thus the maximal concentration of Ni^2+^ ion forming the bicomplex was 100 mM. The value of the NaCl concentration to reach Wr = 1 for Ni^2+^ = 40 mM was about 300 mM, suggesting that there were also monocomplexes of one Ni^2+^ ion and one imidazole ligand, which did not act as crosslinks but had charges.

**Figure 7:**
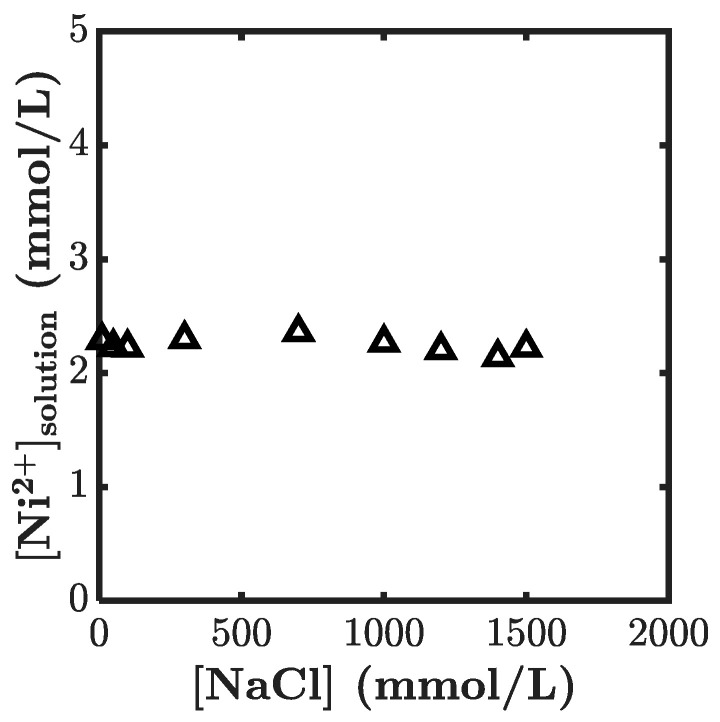
Ni^2+^ concentration in the solution phase at equilibrium binding as a function of NaCl concentration.

Let us briefly comment on the effect of the added NaCl on the coordination complexation. There is no evidence in the literature of the existence of a coordination complex between Na^+^ ions and imidazole ligands. However, the change in ionic strength might have changed the equilibrium of the diffusion–complexation reaction in the polyelectrolyte network. To confirm the absence of the effects of the salt, we carried out diffusion experiments with a fixed initial concentration of nickel ion (Ni^2+^ = 5 mM), and varied the NaCl. As shown in Figure 7, the equilibrium concentration of Ni^2+^ (hence the amount of Ni^2+^ absorbed by the gel) was independent of the amount of NaCl, indicating that the ionic strength and the polymer concentration had no effect on the coordination complexation.

### 2.3. Mechanical Properties of the Dual-Crosslink Gels

#### 2.3.1. Linear Rheology

**Figure 8:**
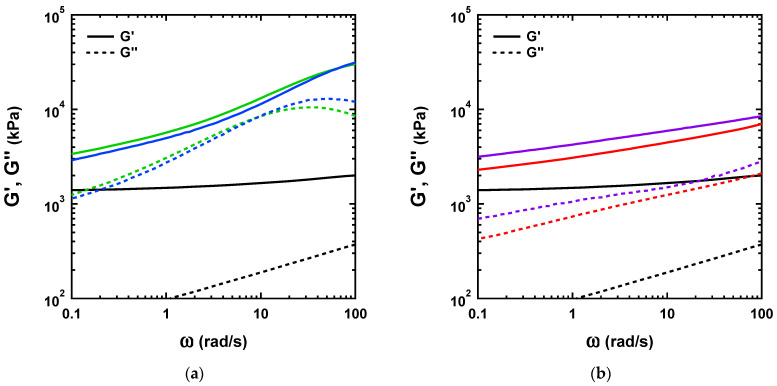
(**a**) Storage and loss moduli as a function of angular frequency for the chemical gel (black), and P(AAm-*co*-VIm)-Ni^2+^ dual-crosslink gels prepared by the one-pot synthesis (blue) and by the diffusion method (green). (**b**) Storage and loss moduli as a function of the angular frequency for the chemical gel (black), and P(AAm-*co*-VIm)-Zn^2+^ dual-crosslink gels prepared by the one-pot synthesis (purple) and by the diffusion method (red).

Here, we compare the gels prepared by both methods at a concentration of Ni^2+^ and Zn^2+^ = 100 mM. Figure 8 shows the results of the small amplitude oscillatory shear experiments in the parallel plate geometry for the dual-crosslink gels physically crosslinked by Ni^2+^ and Zn^2+^, prepared by the two methods. 

As shown in Figure 8a, the rheological response of the chemical gel is characterized by an elastic behavior over the whole frequency range with a value of *G*’ ~ 2 kPa. The moduli for the two P(AAm-*co*-VIm)-Ni^2+^ dual-crosslink gels in Figure 8a exhibited frequency dependences and pronounced values of *G*’’ (peak at about 10 rad/s), due to the dynamic physical crosslinks. While we did not observe a well-defined elastic plateau, at high frequency *G*’ seemed to level off, suggesting the existence of an elastic plateau caused by the associated physical crosslinks. At low frequency, the values of *G*’ approached those of the chemical gel, suggesting that at over a long timescale the physical crosslinks would fully dissociate and the linear rheological properties of the chemical gel could be recovered. The differences in G’ and G” due to the two synthesis methods were small. 

In Figure 8b the dynamic moduli of the P(AAm-*co*-VIm)-Zn^2+^ dual-crosslink gels prepared by the two methods are shown. While the frequency dependence of G’ is clear and qualitatively similar to that of the Ni-based gel, we did not observe a peak in loss modulus, which could have been associated with the lifetime of the physical bonds. This result suggested that the physical bond breaking and chain relaxation occurred at much higher frequencies [13,18,25], which indicated a much shorter lifetime for the Zn^2+^–imidazole bond than that for the Ni^2+^–imidazole bond. At low frequencies, the values of *G*’ approached those of the chemical gel. When the P(AAm-*co*-VIm)-Zn^2+^ gels prepared by the two methods were compared, the same frequency dependences were observed with the same values of tan δ for all frequencies tested (data not shown). The values of the gel moduli prepared by the one-pot synthesis were slightly higher than those of the gel prepared by the diffusion method, presumably due to a slight difference in the Zn^2+^ concentration.

#### 2.3.2. Uniaxial Tensile Tests

**Figure 9:**
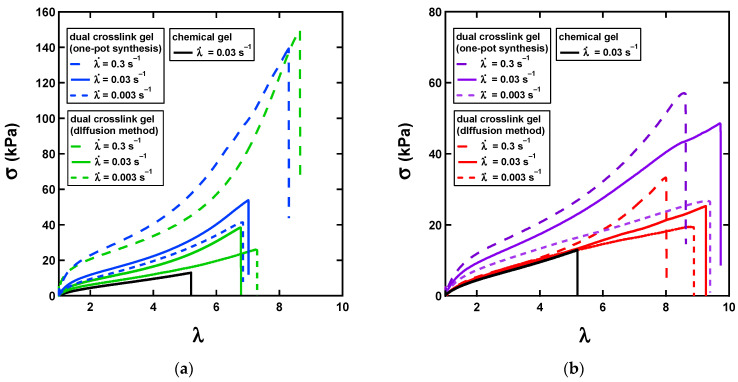
(**a**) Stress–stretch curves for uniaxial tensile tests for the chemical gel (black), and P(AAm-*co*-VIm)-Ni^2+^ dual-crosslink gels prepared by the one-pot synthesis (blue) and by the diffusion method (green). (**b**) Stress–stretch curves for uniaxial tensile tests for the chemical gel (black), and P(AAm-*co*-VIm)-Zn^2+^ dual-crosslink gels prepared by the one-pot synthesis (purple) and by the diffusion method. Dual-crosslink gel (red).

To study the effects of the preparation methods on the mechanical properties under larger deformation, we performed uniaxial tensile tests on the dual-crosslink hydrogels prepared by both methods. Figure 9 shows stress-stretch curves for the [Ni^2+^] and [Zn^2+^] gels measured at three stretch rates and compared to the chemical gel, the behavior of which was neo-Hookean. It had a stretch and stress at break (a sharp drop in stress) at about 4 and at ~10 kPa, respectively, but no stretch-rate dependence was observed (data not shown).

The stress–stretch curves for the P(AAm-*co*-VIm)-Ni^2+^ dual-crosslink gels showed a clear stretch-rate dependence (Figure 9a) for both methods. At the lowest rate (0.003 s^−1^), the initial modulus was close to that of the chemical gel. However, both the value of the stretch at break (to about 7–8.5 kPa) and stress at break (up to 150 kPa) clearly increased, indicating toughness. With an increasing stretch rate, the absolute values of the stress increased as there were more physical bonds that survived at higher stretch rate than at the lower stretch rates (time to reach a given λ was shorter for a higher stretch rate than for a lower). Another important characteristic of the dual-crosslink gel is the strain-hardening behavior observed at high stretch (above about 5) and at a high stretch rate. This strain hardening was much less pronounced at lower stretch rates. We speculated that the non-Gaussian stretching of some of the polymer strands between physical crosslinks caused this strain hardening. In this line of thinking, an increase in stretch rate resulted in an increase in the density of the surviving physical crosslinks, which resulted in an increase in the fraction of chains between the active crosslinks reaching their limiting extensibility, thereby favoring macroscopic strain hardening. The detailed characterizations of the strain hardening will be reported separately in a forthcoming paper.

The results for the P(AAm-*co*-VIm)-Zn^2+^ gel, shown in Figure 9b, were qualitatively similar to those of the P(AAm-*co*-VIm)-Ni^2+^ gel at low stretch rates. The values of the stress were low and comparable to those of the chemical gels. The curve of the gel prepared by the diffusion methods superposed well on the curve for the chemical gel. The stretch-rate dependence and the strain-hardening behavior were less pronounced (note the smaller maximal value of the *y* axis in Figure 9b relative to Figure 9a). This result was consistent with our linear rheology results: the Zn^2+^–imidazole bonds had a shorter lifetime and broke faster than the Ni^2+^–imidazole bonds, and the Zn^2+^-imidazole bonds contributed less to the stress/modulus than did the Ni^2+^–imidazole bonds. Therefore, the stress of the P(AAm-*co*-VIm)-Zn^2+^ gel was similar to that of the chemical gel. The values of the stretch at break were generally higher than those of the P(AAm-*co*-VIm)-Ni^2+^ gel and of the chemical gel: they reached about 9.5, suggesting that the faster exchanging Zn^2+^–imidazole physical bonds efficiently toughened the dual-crosslink network despite the lesser effect on its stiffness. 

When the gels prepared by the two methods were directly compared, there were some interesting differences that illustrated the subtlety of gel synthesis.

The global trend of the stress–strain curves was the same. However, for both P(AAm-*co*-VIm)-Ni^2+^ and P(AAm-*co*-VIm)-Zn^2+^ gels, the one-pot synthesis resulted in slightly higher stress values than the diffusion method. In the case of Zn^2+^, these differences were quite significant. We formulated two hypotheses from this result.

The higher values of stress for the one-pot synthesis, in particular at a low strain rate, may have been due to differences in the chemical network itself, presumably because of the presence of metal complexes during polymerization. While these differences remained small for the Ni^2+^-based gels, they were much more pronounced for the Zn^2+^ gels and modified the interpretation. Conversely, while the dual-crosslink hydrogel prepared by the diffusion method contained NaCl, which partially suppressed the electrostatic interactions, the one-pot hydrogel had no added salt. Thus, the lack of a qualitative behavioral difference between the gels suggested that the electrostatic interactions between the charged physical crosslinking points may have altered the network slightly but did not influence the gel’s mechanical properties much.

## 3. Conclusions

In this paper, we investigated the swelling behavior and mechanical properties of tough polyelectrolyte dual-crosslink hydrogel models synthesized by two different methods: one-pot synthesis and diffusion. In the one-pot synthesis method, polymerization and dual crosslinking occurred simultaneously in a container/reactor, and the resulting gels could be readily used without further swelling for measurements. In this case, the free radical polymerization of the P(AAm-*co*-VIm)-M^2+^ was affected by the presence of the transition metal ions. In particular, we demonstrated that the oxidations of the metal ions by the peroxide radicals resulted in the formation of a metal-oxide precipitate or even in the inhibition of polymerization. However, the majority of the metal ions acted as transient crosslinks when the crosslinker concentration was sufficiently higher than that of the radical initiator. The diffusion method, consisting of preparing first the chemical gel by free radical polymerization in the absence of the metal ions and then incorporating the physical crosslinkers into the chemical gel by diffusion, resulted in dual-crosslink gels at equilibrium swelling. The metal–ligand coordination complexation had two opposite effects on gel swelling: physical crosslinking reduced the degree of equilibrium swelling, while the introduction of additional charges increased it. The charge effect was dominant, and the dual-crosslink gels swelled more than the chemical gel. We showed that the addition of NaCl screened the charge effect and the gel became deswollen to a predefined swelling ratio without influencing the coordination complexation. The comparison of the mechanical properties in the small and large strain showed that the dual-crosslink gels prepared by the two methods exhibited qualitatively similar behavior and comparable toughness. However, the polymerization in the presence of the metal ions did slightly alter the structure of the polymer network and hence its large strain properties, in particular for the Zn^2+^ gel. The presence of charges did not appear to change the dynamics of the bonds and their role in dissipating mechanical energy or in toughening the hydrogels. The results reported here demonstrate the importance of details in the synthesis of chemically crosslinked gels, a process-dependent synthesis that justifies the need for structural characterizations of the dual-crosslink gels. A systematic study of light and X ray scattering was performed on the same gels and will be reported in a forthcoming paper.

## 4. Materials and Methods

### 4.1. Materials

Polyacrylamide (AAm), 1-vinylimidazole (VIm), *N*,*N*’-methylene bisacrylamide (MBA), potassium persulfate (KPS), *N*,*N*,*N*’,*N*’-tetramethylethylenediamine (TEMED), nickel chloride, zinc chloride, sodium chloride, hydrochloric acid, were purchased from Sigma Aldrich (St. Louis, MO, USA) and used as received.

### 4.2. P(AAm-Co-VIm) Chemical Gel Synthesis

Poly(acrylamide-*co*-1-vinylimidazole) chemical gels were prepared by mixing AAm (1.8 M) and VIm (0.2 M) in aqueous solution with MBA (3 mM, corresponding to 0.15 mol% of the monomer units of AAm and VIm) and KPS (6 mM), under nitrogen flow at low temperature (in an ice bath). The solution was then transferred in a glovebox, and TEMED (20 mM) was added. After the overnight crosslinking reaction, the obtained P(AAm-*co*-VIm) chemical gels were used for measurements as prepared.

### 4.3. P(AAm-Co-VIm) Dual-Crosslink Gel Synthesis

P(AAm-*co*-VIm) dual-crosslink gels were prepared by two methods: one-pot synthesis and diffusion.

#### 4.3.1. One-Pot Synthesis

The one-pot synthesis of P(AAm-*co*-VIm)-M^2+^ was performed using the same procedure as the chemical gel, simply by adding extra NiCl_2_ or ZnCl_2_ to the solution. The NiCl_2_ and ZnCl_2_ concentrations were varied between 5 and 100 mmol/L. After the overnight crosslinking reaction, the obtained P(AAm-*co*-VIm)-M^2+^ dual-crosslink gels were used for measurements as prepared.

#### 4.3.2. Diffusion Method

In the diffusion method, three steps were required. First, the chemical gel was prepared as described above (Section 4.2). Then, it was washed of any trace of non-reacted monomers by placing it in at least three baths of distilled water for at least 24 h. At this step, the chemical gel expanded to its equilibrium swelling in water due to the very low chemical crosslinking ratio. Finally, the swollen gel was placed in a solution of NiCl_2_ or ZnCl_2_ to incorporate the metal ions into the chemical gel by diffusion, forming a complex with the imidazole ligands on the polymer chains. The volume of the solution was set equal to 20 times the volume of the gel. Because the Ni^2+^ ions were sensitive to pH (precipitating at pH 7), the pH of the diffusing solution was set at 6.5 with an HCl solution before each experiment.

To investigate the effects of the physical crosslinks on the mechanical properties of the gel accurately, the dual-crosslink gels prepared by diffusion were compared at the same polymer concentration with the dual-crosslink gel and the chemical gel prepared by the one-pot synthesis, the polymer concentration of which was unchanged during the preparation. It should be noted that the complexation of the metal ions induced both deswelling (due to crosslinking) and swelling (due to the introduction of charges to the neutral chemical gel). The swelling ratio was controlled by the addition of NaCl to the system: the electrostatic interactions were screened and the gels deswelled to the same polymer concentration as that of the corresponding chemical gel. For each metal ion concentration, an optimal NaCl concentration was determined. 

### 4.4. Linear Rheology: Small Strain Shear Tests

To characterize the linear viscoelastic properties of the dual-crosslink gels, small-strain oscillatory shear measurements were performed in a parallel plates geometry with roughened surfaces (20 mm in diameter) with an ARES LS1 rheometer (TA instruments). Samples of the gels prepared in sheets were cut into a disk and placed in the parallel plate geometry for gels of both methods. The sample thickness was 1.5 mm. Frequency sweep tests with a dynamic range varying from 0.1 to 100 rad/s were carried out at 25 °C within the linear viscoelasticity regime (0.2–0.8% strain). Note that we used the parallel plate geometry since it was not possible to perform the diffusion method in the geometry due to the volume changes, but it was possible to perform rheological measurements in the geometry of gels prepared by the one-pot method. 

### 4.5. Nonlinear Mechanics: Uniaxial Tensile Tests

The large deformation behavior of the gels was studied by a uniaxial tensile test to fracture and step-cycle loading–unloading tests on an Instron 5565 tensile tester with a 10 N load cell. Samples were rectangular in shape 5 mm wide, 1.5 mm thick, and 15 mm long (between clamps). Following a previously published procedure, we kept the samples in paraffin oil during all the tests to prevent them from drying out [7].

## Data Availability

The data presented in this study are available on request from the corresponding authors.
